# Herodicus: The Father of Sports Medicine

**DOI:** 10.7759/cureus.74224

**Published:** 2024-11-22

**Authors:** Josue Saint-Fleur, Nadiya A Persaud, Latha Ganti

**Affiliations:** 1 Osteopathic Medicine, Orlando College of Osteopathic Medicine, Winter Garden, USA; 2 Research, Orlando College of Osteopathic Medicine, Winter Garden, USA; 3 Emergency Medicine and Neurology, University of Central Florida, Orlando, USA; 4 Medical Science, The Warren Alpert Medical School of Brown University, Providence, USA

**Keywords:** ancient greece, diet and health, greek medicine, herodicus, historical vignette, homeostasis, medical philosophy, physical exercise, preventative medicine, sports medicine

## Abstract

Herodicus is widely recognized as the father of sports medicine. His pioneering approach of integrating physical exercise with medical treatment laid the foundational principles for sports medicine. He began his career as a sports instructor but later pursued medicine, merging his existing expertise in exercise physiology with medical practice. Herodicus believed that poor health arose from an imbalance between diet and physical activity. He emphasized that achieving optimal health required maintaining a balanced diet and engaging in regular physical activity. He applied this holistic approach to patient care, promoting a balanced and harmonious lifestyle as the ideal way to maintain good standards of health. Herodicus’ extensive contributions to sports medicine laid the groundwork for modern medical practice. He advocated a disciplined and balanced lifestyle as the prescription for health and well-being.

## Introduction and background

Early life and medical education

**Figure 1:**
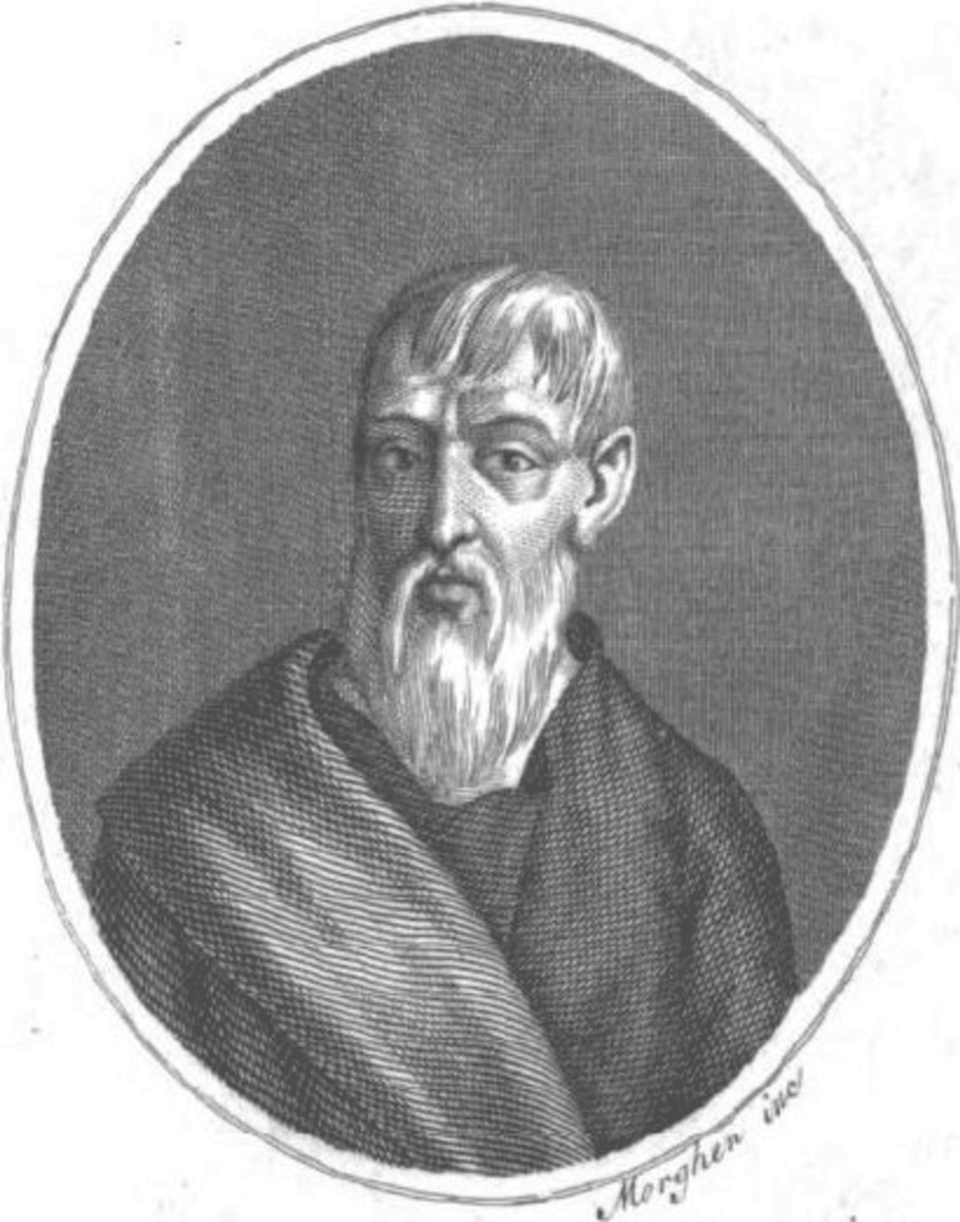
Herodicus Image obtained from Giuseppe Emanuele Ortolani, Public domain, via Wikimedia Commons [4]

Herodicus (Figure 1) was a Greek physician who practiced medicine in the fifth century BC [1]. He was born in Selymbria, an ancient city in Thrace, Greece, that served as a geographical center for medical philosophies, likely influencing his pursuit of medicine [2]. The region’s diverse medical practices are also thought to have shaped Herodicus’ broad and diverse medical ideologies [3].

Prior to embarking on his medical career, Herodicus was a sports instructor where he developed an extensive understanding of physical exercise and how it affects the body, specifically the consequences of its absence [5]. During this time in history, sports instructors prohibited physicians from entering gymnasiums, leading to a widespread lack of understanding of the sports world among medical professionals [6]. This unique perspective and foundational experience laid the groundwork for Herodicus’ later medical philosophies.

In pursuit of medical education, Herodicus studied at Cnidos, one of the most prominent schools in ancient Greece, where his education was rooted in the teachings of Hippocrates [7]. Hippocrates, often regarded as the Father of Medicine, promoted a holistic approach to medicine emphasizing lifestyle, environment, and diet as key contributors to good health [7]. This philosophy aligned with Herodicus’ belief that both exercise and nutrition were fundamental in maintaining optimal health [1]. At Cnidos, the curriculum emphasized the importance of obtaining detailed, comprehensive patient histories during patient encounters and developing differential diagnoses [8]. Herodicus integrated these teachings into his medical practice, combining them with recommendations for a strict diet, consistent physical activity, and regular training as effective treatment methods [5].

Even with minimal written records from that period, we can trace the impact Herodicus has had in laying down the foundations for modern sports medicine. This paper will explore how Herodicus pioneered sports medicine, his impact on future practitioners in the field, and his legacy left behind in sports medicine practice.

## Review

Concept of physical exercise

Herodicus, best known for his pioneering advocacy for physical exercise as a means of treating and preventing disease, argued that exercise had a multitude of both preventative and therapeutic benefits for the human body [9]. The two primary advantages he suggested were that exercise could strengthen the body and expedite recovery from illness [7]. This theoretical perspective stems from Cnidos founder and Herodicus' mentor, Euryphon, who believed that diet played a crucial role in maintaining health [8]. Herodicus expanded on this idea, postulating that food cannot be properly digested without corresponding motion [7]. He believed that improper digestion could produce by-products, yielding two types of bodily fluids: sharp and bitter. The resulting disease arising from these fluids was dependent on which type of fluid was most prevalent and where it accumulated in the body [10]. The sharp and bitter bodily fluids described by Herodicus are most similar to what later medical theories would identify as phlegm and bile [9,10]. Herodicus stressed the importance of physical activity, asserting that sufficient physical activity and a proper diet were vital in maintaining good standards of health, building physical resilience and reducing susceptibility to disease [10-13].

Early theories of homeostasis

Herodicus developed his own medical system, which emphasized a strict diet focused on grains and vegetables, which were highly nutritious. This diet was paired with a rigorous training regimen that included running, weight training, and other forms of resistance exercises [2]. Although Herodicus received criticism for his intense medical ideologies and his incorporation of physical activity into medicine, he pioneered what we call modern sports medicine today [8]. Herodicus’ theory of the homeostasis of two liquids later influenced Hippocrates’ theory on the homeostasis of the four humors [9,14].

Criticism of Herodicus

Although innovative, Herodicus’ medical system faced criticism from others. Among his critics was his own student, Hippocrates, who argued that Herodicus’ prescribed exercises were excessively strenuous and, at times, impractical for specific groups of patients [15]. Herodicus was also criticized for recommending exercise routines for acutely ill patients, including those with fevers or recovering from injuries [14]. Herodicus strongly believed that exercise and diet were universal remedies for most diseases, leading him to consistently advocate for high-intensity exercise for his patients, even when a more moderate approach may have been necessitated by their condition. Although rigorous physical activity was central to his medical philosophy, some of his exercise prescriptions may have overlooked the unique needs and limitations of individual patients, potentially posing greater risks than benefits [2]. This idea resonates in modern-day medicine, where we now recognize the importance of tailoring treatment plans and exercise regimens to each patient’s unique limitations and needs.

Legacy, influence, and contributions to sports medicine

Herodicus recognized the detrimental, atrophying effects physical inactivity had on the human body [16]. He postulated the first systems of exercise, coining the term “the art of gymnastics” [17]. He pioneered medicinal gymnastics, a system of exercise that used wrestling, walking, and massage as therapeutic methods to treat physical ailments, restore health, and maintain bodily balance [16,18]. As Herodicus observed the health benefits experienced by young athletes, he adapted concepts from athletic and military gymnastics to create structured exercise regimens to improve overall health. He tailored these regimens by factoring in age, sex, climate, illness, and diet, a newly emerging field in medicine [18]. This knowledge was shared and incorporated into ancient Greek and Roman medicine, where prescriptive exercise, massage, joint mobilizations, and hydrotherapy were used to keep various humors in balance [9]. Herodicus’ ideas greatly influenced Hippocrates and subsequent physicians, who embraced medicinal gymnastics as an essential aspect of medical practice, setting the foundation for what is now recognized as sports medicine [18].

## Conclusions

Herodicus’ pioneering contributions to medicine, specifically integrating physical exercise with medical treatment, laid the foundational principles for modern-day sports medicine. His background as a sports instructor proved invaluable; he applied his prior knowledge, coupled with extensive medical training, to formulate an innovative approach to health that emphasizes the critical balance of diet and exercise, highlighting the importance of maintaining bodily homeostasis. His monumental theories on the interactions and interconnections between diet, exercise, and bodily fluids have profoundly influenced medical practices and shaped modern medicine. His concept of medicinal gymnastics illustrated the therapeutic powers of exercise, establishing the fundamentals of preventative care within sports medicine. Today, modern sports medicine continues to reflect Herodicus’ foundational concepts, promoting exercise and balanced nutrition as essential for maintaining overall health. Herodicus’ impactful work resonates with the principles of modern preventative medicine. He advocated that consistent daily habits, such as proper nutrition and regular exercise, were key contributors to sustaining health. He believed that upholding these principles in day-to-day life could prevent illness, maintain health, and reduce the need for medical intervention. Although sports medicine continues to evolve, its core principles remain rooted in Herodicus’ philosophy of prescribing exercise and a balanced diet for a healthy lifestyle. Herodicus’ enduring legacy laid the foundation for sports medicine, defining core concepts that remain integral to modern health and well-being.

## References

[1] Echenberg D (2007). [A history of internal medicine: medical specialization: as old as antiquity]. Rev Med Suisse.

[2] Burnet J (1901). Plato's works, vol. 2: tetralogy III-IV (Book in Latin). https://www.oxfordscholarlyeditions.com/display/10.1093/actrade/9780198145417.book.1/actrade-9780198145417-book-1.

[3] Jouanna J (1974). Hippocrates. For an archeology of the School of Cnidus (Book in French).

[4] (2017). File:Herodicus.png. https://commons.wikimedia.org/wiki/File:Herodicus.png.

[5] Georgoulis AD, Kiapidou IS, Velogianni L, Stergiou N, Boland A (2007). Herodicus, the father of sports medicine. Knee Surg Sports Traumatol Arthrosc.

[6] Cohen MR (1945). A source book in Greek science. Harvard University Press.

[7] Dambasis IN (1966). Historikai latrikai Meletai Seira Proti.

[8] Adler A (1928). Suidae lexicon. https://archive.org/details/adler-a.-suidae-lexicon-3-./Adler%20A.%2C%20Suidae%20Lexicon%201%20%28%CE%91-%CE%93%29/page/494/mode/2up.

[9] Sigerist H (1987). A history of medicine. A history of medicine.

[10] Diels H (1893). Anonymus of London from the eclogue of Aristoteles iatricis Menonii and other physicians. Greek commentaries on Aristotle (Book in Latin). Supplementum Aristolicum.

[11] Longrigg J (1993). Greek rational medicine: philosophy and medicine from Alcmaeon to the Alexandrians.

[12] Burnet J (1903). Plato's works, vol. 3: tetralogy V-VII (Book in Latin). https://www.oxfordscholarlyeditions.com/display/10.1093/actrade/9780198145424.book.1/actrade-9780198145424-book-1.

[13] Burnet J (1902). Plato's works, vol. 4: tetralogy VIII (Book in Latin). https://www.oxfordscholarlyeditions.com/display/10.1093/actrade/9780198145448.book.1/actrade-9780198145448-book-1.

[14] Edelstein L (1987). Ancient medicine: selected papers of Ludwig Edelstein. Ancient Medicine..

[15] Hartofilakidis-Garofalidi Hartofilakidis-Garofalidi, Papathanassiou BT (1972). Orthopaedics in ancient Greece. Clin Orthop Relat Res.

[16] Grensemann H, Knidische Medizin, Teil I (1975). The testimonies on the oldest Knidian teaching and analyzes of Knidian writings in the Corpus Hippocraticum (Article in German). Ars Medica II.

[17] Schumacher J (1963). Ancient medicine: the natural-philosophical foundations of medicine in ancient Greece (Book in German). Antike Medizin.

[18] Jaucourt L (1757). Medical gymnastics (Book in French). https://quod.lib.umich.edu/d/did/did2222.0003.956/--medicinal-gymnastics?rgn=main;view=fulltext.

